# Supplementary material for: XLLC-Net: A lightweight and explainable CNN for accurate lung cancer classification using histopathological images
Source: PLoS One. 2025 May 30;20(5):e0322488. doi: 10.1371/journal.pone.0322488 (PMC12124586; doi:10.1371/journal.pone.0322488)
Supplement: Attachment — Submitted filename: Response to Reviewers PLOS ONE.pdf [file pone.0322488.s001.pdf]

Journal: PLOS ONE
Manuscript ID: PONE-D-24-32864
Title: XLLC-Net: A Lightweight and Explainable CNN for Accurate Lung Cancer Classification Using Histopathological Images
Authors: Jamin Rahman Jim, Md. Eshmam Rayed, M. F. Mridha, Kamruddin Nur.

Dear Editor in Chief/Reviewers,

We would like to thank anonymous reviewers for their valuable comments and the editor for his acceptance to revise our paper and for their specific and important comments. We have revised the paper and restructured several sections. All changes in the paper are presented in the updated version.

Response of Reviewer-1

Reviewer Concern-1: Firstly, the paper is written inconsistently, with numerous repetitions and phrases that seem to originate from automated text generation tools, such as chatbots. While this isn't inherently negative, thorough editing is necessary after the initial draft to enhance clarity and cohesion.

Author's Response: Thank you for your valuable feedback. We appreciate your suggestions.

Author's Action: We have edited our manuscript to enhance clarity and cohesion.

Reviewer Concern-2: The authors present a conventional CNN architecture that consists of convolutional layers, batch normalization, max-pooling, and dropout layers, repeated four times. This design lacks novelty, as it adheres to a typical structure found in many existing models. Additionally, while the authors compare their model with some state-of-the-art architectures, these comparisons involve models developed for different tasks (see Table 5), which may not provide a valid benchmark.

Author's Response: We appreciate the reviewer's feedback and understand the concern regarding the novelty of our CNN architecture. Our primary goal was to explore whether a lightweight network could effectively perform the given task, and our results demonstrate that it performs exceptionally well. While the structure of our model follows standard CNN principles, its effectiveness in achieving superior results with reduced complexity is a key contribution, particularly for resource-constrained environments.
Regarding the benchmark comparisons in Table 5, we would like to clarify that all the state-of-the-art models—AlexNet, VGG-17, VGG-19, and ResNet-50—were trained on the same LC25000 dataset for a fair and valid evaluation. Comparing the performance of a newly proposed model against widely used deep learning architectures trained on the same task is a standard practice in the field. This allows for a meaningful assessment of our model's efficiency and accuracy.

Author's Action: Since these clarifications were already included in the manuscript, no modifications were made. However, we remain open to further suggestions if additional elaboration is required.

Reviewer Concern-3: On page 9 (Table 6), they mention more advanced models trained specifically for medical image analysis, yet they do not directly compare their model against these in terms of the number of parameters.

Author's Response: Thank you for pointing out the need for a direct comparison of model parameters. We appreciate this suggestion and have incorporated it into our revised manuscript.

Author's Action: We have added a "Total Params (M)" column in Table 6, including parameter counts for models where available. For models that did not report parameters in their original papers, we have marked them as "N/A" to maintain accuracy.

Reviewer Concern-4: Furthermore, they fail to present standard deviations or disclose the number of trials (i.e., initialization) conducted, which are important for assessing the reliability and generalization of their results. As the differences in the metrics are very small, it is important to re-run the experiments at least 5 times per model.

Author's Response: We appreciate the reviewer's suggestion regarding the importance of multiple trials to assess the reliability and generalization of our results. To address this, we have conducted five independent training trials of the XLLC-Net model and reported the corresponding accuracy, precision, recall, and F1-score for each trial. Additionally, we have now included the mean and standard deviation of these performance metrics to provide a comprehensive understanding of the model's stability and robustness.

Author's Action: We have updated the manuscript to explicitly mention that the model was trained five times, and we now present the results for each trial in Table 4. The table includes accuracy, precision, recall, and F1-score values across all trials, along with the mean $\pm$ standard deviation to quantify variability. Additionally, we have revised the Results Analysis section to emphasize the consistency of our model across multiple runs, ensuring its reliability and generalization capabilities.

Reviewer Concern-5: The authors do not present and mention anything about how they have chosen the hyperparameters. For example, dropout rates, number of epochs, architecture (number of layers, number of nodes on the fully connected layers, etc).

Author's Response: We appreciate the reviewer's insightful comment regarding hyperparameter selection. To address this, we have added a dedicated explanation in the Methodology section, explicitly detailing the rationale behind key hyperparameters, including dropout rates, number of epochs, network architecture, batch size, optimizer choice, and loss function. These decisions were made using standard deep learning techniques for medical imaging tasks.

Author's Action: We have included a "Hyperparameter Selection" subsection in the Methodology section, where we provide a clear justification for each parameter choice.

Reviewer Concern-6: Use the abbreviation defined for later references to the same phrases (for example, deep learning (DL) is defined in line 8, and then the full phrase is used again in line 83). Line 101, 110: Convolutional neural networks $\rightarrow$ CNNs

Author's Response: Thank you for highlighting the inconsistency in abbreviation usage. We appreciate this suggestion and have carefully revised the manuscript to ensure consistency.

Author's Action: We have standardized all abbreviations, replacing Machine Learning with ML, Deep Learning with DL, Convolutional Neural Networks with CNNs, and Explainable AI with XAI throughout the manuscript. The corrected terms are marked in blue in the "Revised Manuscript with Track Changes" file.

Reviewer Concern-7: Line 139: "Cibi et al. presented a customized deep CNN model using CapsNet [...]": I do not understand what the "CapsNet" is, it is not defined in the paper.

Author's Response: Thank you for highlighting the missing definition of CapsNet. We appreciate this feedback and have now incorporated a brief explanation of Capsule Networks (CapsNet) in the manuscript to ensure clarity.

Author's Action: We have revised the sentence to include a short explanation of CapsNet before its first mention in the manuscript.

Reviewer Concern-8: Methods (equations): In Eq. 12 the authors give the summation from j=1 to 3, however in Eq. 14 they use a more generic form using C to denote the number of classes. I suggest changing the Eq. 12 to denote the summation from j=1 to C and give below the explanation of C.

Author's Response: Thank you for your suggestion regarding consistency in notation. We appreciate this feedback and have modified Equation 12 to use a generic notation C instead of the fixed number 3, ensuring uniformity with Equation 14.

Author's Action: We have updated Equation 12 to denote the summation from j=1 to C and added an explanation stating that C represents the total number of classes in the classification task.

Reviewer Concern-9: Figures 5-8: The y-axis should be the same across all subplots to ensure fair comparison with visual inspection.

Author's Response: Thank you for your observation. We appreciate your suggestion regarding the y-axis consistency across subplots.

Author's Action: The figures are automatically generated from the code, and while there are slight differences in the y-axis scale, they do not impact the accuracy of the presented results. Each subplot correctly reflects the training and validation trends for the respective models, ensuring a fair comparison. Given the computational complexity and resource constraints, re-running all models to adjust the y-axis is not feasible at this stage. However, the trends remain clearly interpretable despite this minor variation.
